# Oceanic and ionospheric tidal magnetic fields extracted from global geomagnetic observatory data

**DOI:** 10.1098/rsta.2024.0088

**Published:** 2024-12-02

**Authors:** Robert H. Tyler, David S. Trossman

**Affiliations:** ^1^ NASA Goddard Space Flight Center, Planetary Magnetospheres Laboratory, Greenbelt, Maryland, MD, USA; ^2^ Earth System Science Interdisciplinary Center, University of Maryland at College Park, College Park, MD, USA

**Keywords:** ocean tides, ionospheric tides, geomagnetic fields, ocean heat content

## Abstract

Ocean tide generated magnetic fields contain information about changes in ocean heat content and transport that can potentially be retrieved from remotely sensed magnetic data. To provide an important baseline towards developing this potential, tidal signals are extracted from 288 land geomagnetic observatory records having observations within the 50-year time span 1965–2015. The extraction method uses robust iteratively reweighted least squares for a range of models using different predictant and predictor assumptions. The predictants are the time series of the three vector components at each observatory, with versional variations in data selection and processing. The predictors fall into two categories: one using time-harmonic bases and the other that directly use lunar and solar ephemerides with gravitational theory to describe the tidal forces. The ephemerides predictors are shown to perform better (fitting more variance with fewer predictors) than do the time-harmonic predictors, which include the traditional ‘Chapman–Miller method’. In fitting the oceanic lunar tidal signals, the predictants with the highest signal/noise involve the ‘vertical’ magnetic vector component following principle-component rotation. The best simple semidiurnal predictor is the ephemeris series of lunar azimuth weighted by the inverse-cubed lunar distance. More variance is fitted with predictors representing the lunar tidal potential and gradients calculated for each location/time.

This article is part of the theme issue ‘Magnetometric remote sensing of Earth and planetary oceans’.

## 1. Introduction

Coupling of the electrically conducting ocean with Earth’s magnetic field causes a small amount of the ocean’s tidal-flow energy to be converted to electric currents, with associated magnetic fields that reach through and even far outside the ocean. While it is not expected that the energy transfer rate is high enough to be dynamically important in the tidal-flow energy budget, the tidally generated electric and magnetic fields are of oceanographic interest because observations of these fields may be used to diagnose and monitor the ocean tidal process.

**Figure 1.**
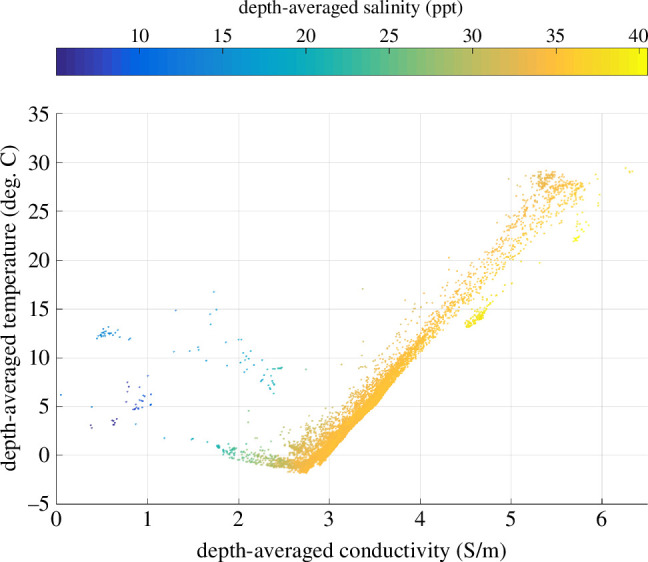
A global, gridded climatological database of ocean conductivity derived through objective analyses of temperature, salinity and depth observations shows a remarkable correlation between depth-averaged temperature and depth-averaged conductivity, suggesting that OCC might be used as a proxy or constraint on OHC. The colour coding shows that the data points that diverge from the correlation are from regions that have anomalous salinity, such as inland seas. (Redrawn from [1].)

A motivation of this study has been the proposal for using modulations in the observed tidal magnetic signals to describe changes taking place in the ocean’s tidal response and temperature-dependent electrical conductivity, as both of these are expected to change with heat uptake by the ocean [1–6]. In the construction and analysis of the first climatological database for global ocean conductivity, it was found that while conductivity varies with pressure, temperature and salinity, the depth integrated conductivity (i.e. ‘ocean conductivity content’ (OCC)^1^ is remarkably well correlated with depth-integrated ocean heat content (OHC), figure 1). Because approximately 90% of the Earth’s heat uptake owing to climate change has gone into the ocean, which is severely under sampled *in situ*, the development of remote-sensing methods for monitoring OHC is an active area of research; OCC can potentially be monitored or constrained using magnetometric remote sensing of the ocean tidal magnetic fields. In the simplest conception, observed modulations in the tidal magnetic fields would reveal modulations in OCC and, by proxy, OHC. In more sophisticated approaches, the magnetic fields and/or OCC provide constraints that can be used or assimilated in models of the ocean state [6,8–10].

In assessing this potential, it is important to appreciate that the oceanic tidal magnetic signals are relatively weak and share time and space scales with other magnetic sources. As such, the accuracy and precision at which these signals can be separated are critical. Tidal magnetic fields are generated by electric currents in the ocean as well as the ionosphere/magnetosphere, and there have been early and continuing studies to separate the magnetic fields from the oceanic and ionospheric sources (for modern reviews that include this material, see [11–13]). While early work [14–27] was mostly focussed on removing the confounding oceanic contribution such that the ionospheric component could be better isolated, the primary goal in this study is rather to best isolate the oceanic contribution for the reasons described above. The magnetic fields generated by the oceanic and ionospheric tides have overlapping spatial and temporal frequencies but there are also important differences in their behaviour that have been exploited in their separation. From an Earth-fixed frame, the tidal forces propagate westward as the Earth spins with respect to the Sun and Moon. The propagation of the ocean’s response is, however, constrained by the ocean basins. The magnetic fields generated by the ocean tides depend on the tidal-flow velocity, the ocean conductivity, the ambient main magnetic field and, to a lesser extent the conductivity of the sediments, continents and mantle. By contrast, tides in the ionosphere propagate westward more freely but the electrical conductivity and magnetic fields generated are weak at night because the conductivity depends on ionizing solar radiation. To some approximation, then, the ocean tidal fields are active day and night while the ionospheric tidal fields are active only during the day. This behaviour is used in the traditional ‘Chapman–Miller method’ for separating oceanic and ionospheric lunar tidal contributions in data from land geomagnetic observatories. This method has shown some early success in describing the ionospheric magnetic fields using land observatory data, but there was little resolution of the oceanic component. Not only was resolving the magnetic fields due to the global ocean tides not the goal in early work, but there was little opportunity for doing so with such sparse coverage from the land observatories and little guidance on what to expect. Hence, while it has been long-since known that there are ocean tidal components in the geomagnetic observatory data, there was not in the early work a description or prediction of the global distribution of the fields.

Numerical forward models and modern low-orbit satellite magnetic surveys have revolutionized the description of ocean tidal magnetic fields. While the satellite magnetic data describe spatial coverage of the tidal magnetic field that was not previously available, the hourly records of the global geomagnetic observatories recorded through decades and even over a century provide the best estimates, at least at the locations of the observatories, as well as temporal coverage for examining ocean tidal variability in the past.

Importantly, the gravitational tidal forces can be calculated with very high accuracy for the present, past and future from astronomical ephemerides of the Moon, Sun and gravitational theory. Accounting for changes in the forces, the variations in the ocean tidal signals then describe variations in the ocean response parameters. Because of the predictability of the tidal forces and the near stationarity of the ocean response parameters, the ocean tides are also quite regular. It appears, however, that the ocean tidal response parameters have in fact been changing over time, and this has become a topic of high interest because of the implications of climate change on ocean parameters. Changes in the tidal response have primarily been seen in tide-gauge data, but recently tidal response modulations over time have also been recovered from geomagnetic data from the Honolulu observatory [28], and this has motivated work in progress to systematically extract the tidal modulation series from records of the full global set of geomagnetic observatories. The scope of the work in this study does not include recovery and description of the modulations over time of the tidal magnetic fields.

Rather, this study aims to create, through a systematic line-of-data processing, a baseline dataset of the tidal amplitude and phase at the global set of geomagnetic observatories. To create tidal estimates with high confidence as well as the largest geographic distribution; all observations within the 50-year time span of 1965–2015 are included in the analyses.

A related goal of this study is to explore and compare regression methods for extracting the tidal signals using as predictors either the customary time-harmonic bases of the constituent frequencies, or bases derived from ephemerides and gravitational potential theory. In a specific comparison (between the time-harmonic constituent $M_{2}$ and the lunar ephemeris base it approximates) where results are expected to be similar, this is found (e.g. figure 8). Furthermore, results from many observatories show high confidence in the tidal fits using either approach. But this agreement is mostly a result of admitting data from such a large time span. Indeed, a recent study [29] extracting tidal signals from a much smaller time-window of the geomagnetic data showed low confidence, with standard errors often rivalling the amplitudes of the estimated coefficients. The small time-window in [29] was chosen to coincide with a period of geomagnetically quiet conditions, when less noise might lead to better confidence in the tidal fits. Additionally, the lack of nodal corrections in the specific time-harmonic regression method used in [29] would also limit accuracy in the fits if time series longer than approximately a year were used. Here, we find much higher confidence using a large time-window of data. This option is less available for studies aimed at resolving tidal modulations because the temporal resolution in the results is limited by the time-window used in the analyses.

In this paper, we focus on describing the lunar semidiurnal tide. Results from the larger set of models studied as well as details on data processing and tutorial material are included in appendix A.1.

## 2. Magnetic field as a function of lunar and solar azimuth

The goal motivating the separate analyses for night and day data is to separate the oceanic and ionospheric tidal signals, and this approach has been extended from early studies of observatory series up to modern studies involving satellite magnetometer data. Indeed, this approach is also followed below to produce the main results for the tidal fits for the global set of observatories. Because we provide results for both night and day data, inferences about the differences in behaviour of the ocean and ionospheric tidal fields can be made but are limited by the simple two-category assumption. The approach in this section provides more information on these differences, and provides a method for examining the appropriateness of the night/day criterion assumed for a specific observatory dataset.

Here, we explore representation of the night + day data as functions of lunar and solar azimuth coordinates representing the Earth-frame longitude position of the Moon and Sun. In this case, there is no night/day data selection nor removal of the solar daily signal. The data treated are simply the vector geomagnetic series (eastward E, northward N, radial R) with centred time differencing and then detrending applied: $\dot{E}$, $\dot{N}$, $\dot{R}$. Data during times when the disturbance index $K_{p}>2$ are removed. The data are fitted to a grid with 2° azimuthal resolution using Barnes interpolation with cyclic boundary conditions. A second smoothing iteration uses the results from the first iteration as input. The smoothing distance parameter for the Gaussian weighting is isotropic and set to 10° for the first iteration and 15° for the second.

**Figure 2.**
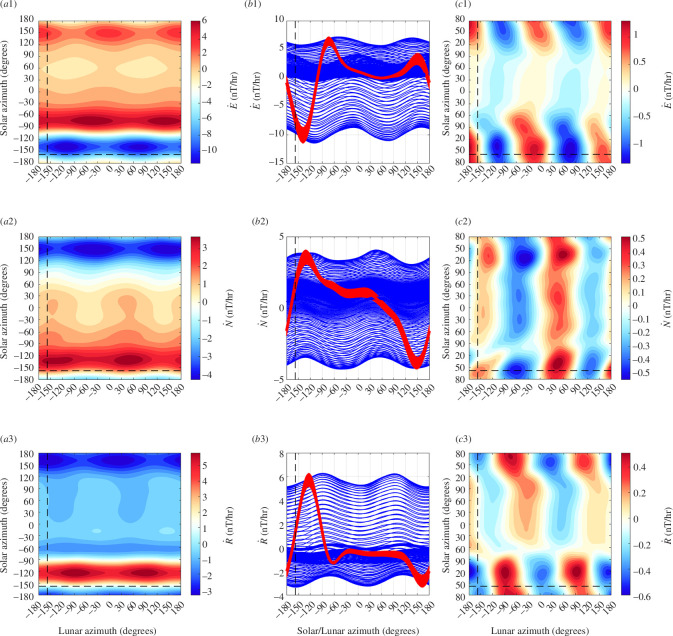
Honolulu magnetic data ($\dot{E}$, $\dot{N}$, $\dot{R}$, with $K_{p}>2$ data removed) is interpolated on to a regular grid to show the respective data (frames *a*1–*a*3) as functions of lunar and solar azimuth (longitude) position. The same data plotted against each axis (*b*1*–b*3) clearly shows that solar daily dependence (red) dominates lunar (blue). When the mean solar curve is removed from *a*1*–a*3, the result is *c*1–*c*3 which better shows the remaining lunar semidiurnal waveform. Dashed lines are drawn at the longitude of the observatory and indicate the phase at which the Moon or Sun is overhead. Note that the lunar waveform phase varies with solar azimuth and is stronger during the day, indicating that the lunar tidal magnetic fields depend on both the position of the Moon and the Sun.

**Figure 3.**
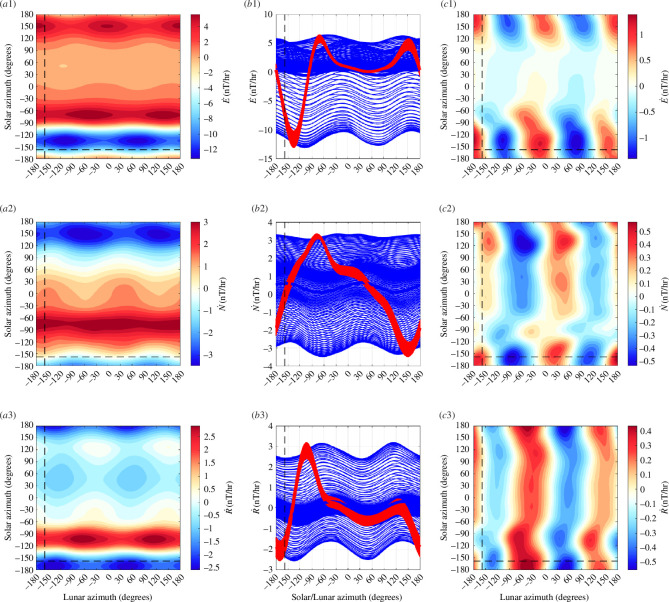
Similar to figure 2 but for data with PCA rotation. Note the reduction in the solar influence on the lunar waveform in *c*3.

The solution for the Honolulu geomagnetic observatory data is shown in figure 2. The dominant feature is a solar-daily waveform that does not follow a simple semidiurnal sinusoid as would be expected from the Sun’s gravitational tide. Instead, the waveform with larger amplitudes during day time are due to solar radiation. When the mean solar waveform is removed, a semidiurnal lunar waveform prevails but still shows a dependence of amplitude and phase on time of day. As found in [28], the separation of the two lunar contributions can be improved by first rotating the vector data to its principle components using principle component analysis (PCA). A similar figure but for the PCA rotated data is shown in figure 3. Despite that the rotated ‘vertical’ component is only rotated 27° from the geographic vertical, the lunar waveform shows much less dependence on solar azimuth. The interpretation here is that the rotated vertical component has become dominated by the oceanic tide by avoiding the principle axes of external fields (which would be horizontal only in the approximation of these sources as horizontal sheet currents).

**Figure 4.**
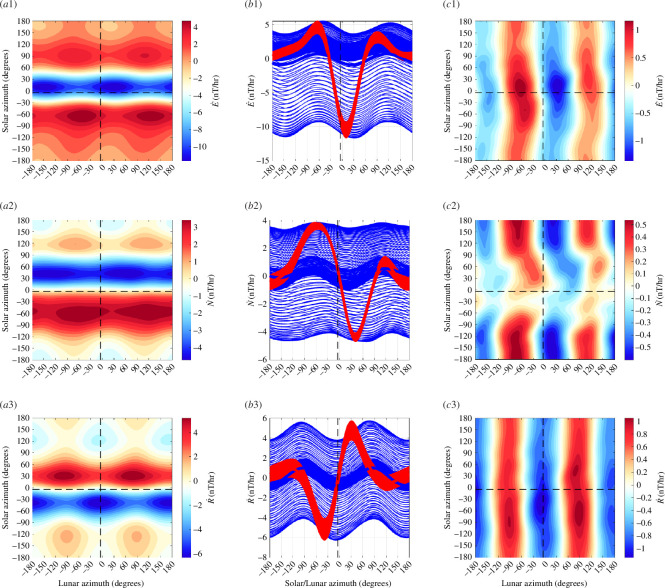
Similar to figure 2 but for data from the HAD observatory where ocean tidal magnetic fields are stronger.

**Figure 5.**
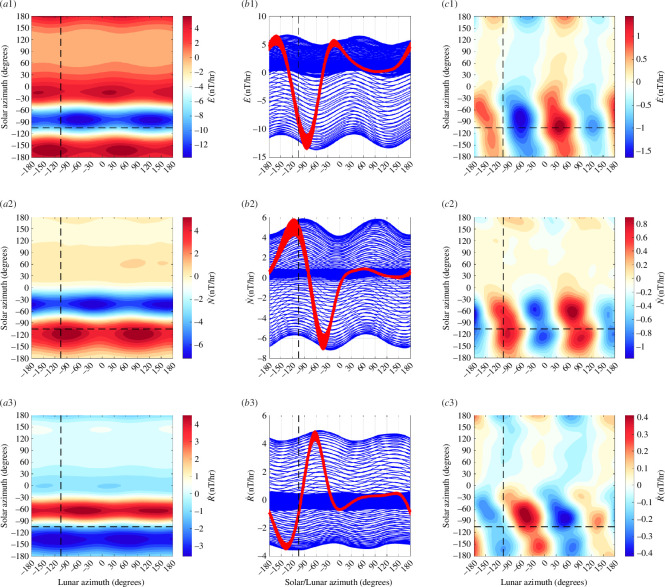
Similar to figure 2 but for data from the Boulder observatory where oceanic tidal magnetic fields are weaker.

For comparison, we present similar figures for other observatories. An observatory with larger ocean tidal influence than Honolulu is Hartland (HAD) in the United Kingdom (figure 4), and with weaker oceanic influence is the observatory at Boulder (BOU) in the United States (figure 5). As should be expected, we see a stronger lunar semidiurnal waveform for HAD and a weaker form for BOU. Indeed, ocean tides clearly dominate ionospheric tides during both night and day at HAD, whereas the opposite may be true at BOU. PCA rotation improves the waveform for the rotated ‘radial’ component (rotated by 21.3° for BOU, and 15.3 for BOU) in each case. PCA rotation also improves the oceanic signal-to-noise ratio not only in these individual cases but also, at least statistically, in the global set of observatories. The latter will be seen in the regression results (presented later) where we find on average higher statistical significance in the tidal fits of the rotated vertical component compared to the unrotated.

## 3. Observed lunar semidiurnal magnetic fields

We show here results for the lunar semidiurnal tidal coefficients extracted from the magnetic series, specifically, the subset of series from observatories where the fits were statistically significant (*p*‐value ≤ 0.05). We first describe results using the traditional time-harmonic bases and then compare with result using lunar ephemeris bases. Specifically, we compare case 2 and model 3 from the collection of regression results described in detail in appendix A.

While the lunar semidiurnal variation has a clear and compact representation using ephemeris coordinates and gravitational theory (the semidiurnal component of the lunar tidal potential appears as the sine and cosine of lunar azimuth scaled inversely by lunar distance cubed), the traditional time-harmonic representation is spread through a series of constituents. The dominant constituent, $M_{2}$, can be described as approximating the lunar orbit as steady, circular and equatorial. Constituents $N_{2}$, $L_{2}$ can be thought of as corrections for the orbit’s ellipticity and faster motion at perigee. Further constituents account for lunisolar coupling and long period variations in the orbit. It suffices to describe here results for the stronger $M_{2}$ constituent.

**Figure 6.**
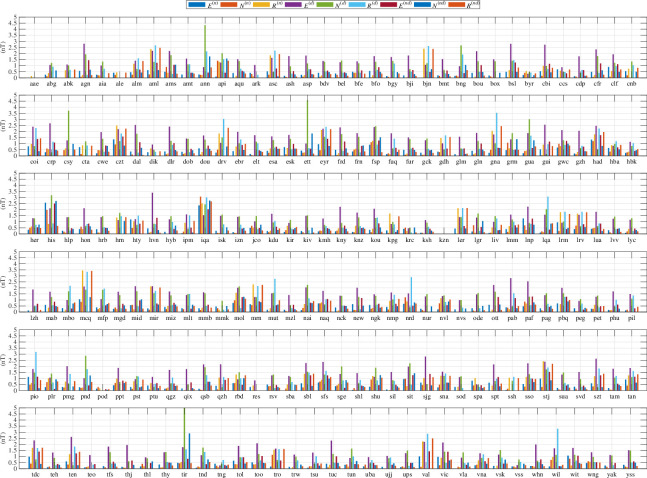
Amplitude of the $M_{2}$ tide extracted from geomagnetic observatory data using time-harmonic bases. For each observatory, there are nine estimates representing the three vector component series (*E*, *N*, *R*) using only nighttime data (n), daytime data (d) or night and day data (nd).

**Figure 7.**
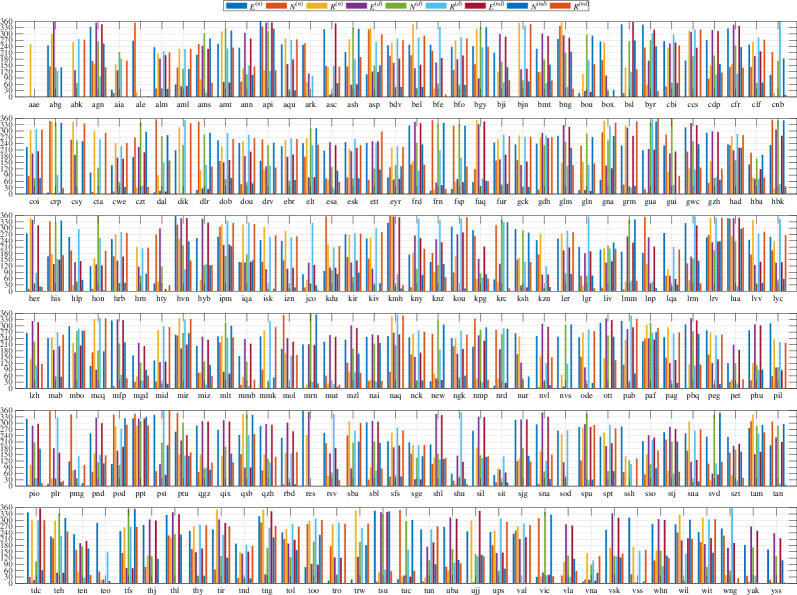
Phase (degrees) of the $M_{2}$ tide extracted from geomagnetic observatory data using time-harmonic bases. For each observatory, there are nine estimates representing the three vector component series (*E*, *N*, *R*) using only nighttime data (n), daytime data (d) or night and day data (nd).

**Figure 8.**
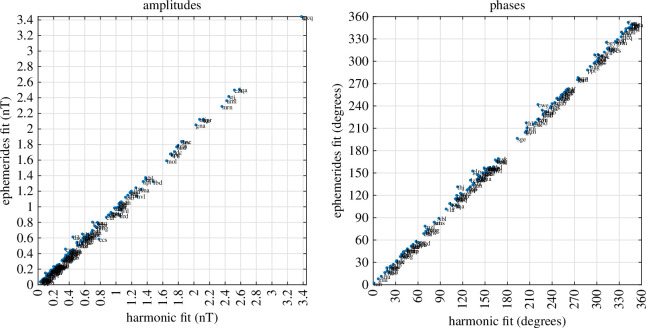
Scatter plots for the amplitude and phase of the nighttime (radial component) $M_{2}$ tidal fits at each observatory obtained using either time-harmonic or lunar ephemeris bases show very good agreement.

While the $M_{2}$ time-harmonic bases do not capture as much tidal energy as the ephemerides/potential-derived bases, much of the discussion of ocean tidal magnetic fields in observations and modelling has been focussed on $M_{2}$. This time-harmonic base is not required to extract tidal signals from the data but it is required in the time-harmonic numerical forward models that predict the magnetic field. Hence, a description of the observed $M_{2}$ constituent is important for multiple reasons. To compensate for the time-differencing and obtain the coefficients reflecting the original (without time differencing) series, we translate the amplitude/phase coefficients to complex form, divide by −iω, where $i=\sqrt{-1}$ and ω is the constituent ($M_{2}$) frequency, then convert again to amplitude and phase. The estimates from the time-harmonic fit are shown in figures 6 and 7. The estimates from the ephemeris fit are very similar and are shown in figures 19 and 20. The similarity is summarized in figure 8, which also shows that the additional variance fit by the lunar ephemeris is relatively small (i.e. $M_{2}$ is a good approximation to the lunar semidiurnal variation.) Overall, confidence in either of the estimates tends to be high (zero *p*‐value) because most of the observatories provide much data within the 50-year window. By comparison, confidence levels in the estimates described in [19] or [29] are much lower because of the much narrower time spans of data used. Using longer time spans of data in these earlier studies fitting simple time harmonics that do not correct for nodal precession of the lunar orbit is problematic. In this study, nodal precession is either corrected for (UTide) or circumvented (using ephemerides).

We attempt now to describe the separate parts of the $M_{2}$ tidal signals in the geomagnetic observatory records that are due to tidal processes in the ocean versus those in the ionosphere. The two processes create fields that overlap in frequency and spatial pattern and so the best approach for this separation is not obvious. A distinction between the two is that the ocean lunar tidal magnetic fields are active both at night and during the day, whereas the ionospheric tidal magnetic fields are mostly active during the day (the solar ephemerides are used to calculate the angle from solar zenith at each observatory; ‘night’ data are prescribed as those with zenith angle greater than 100°, and ‘day’ is prescribed as with zenith angle less than 80°). We may then use our results to estimate the ionospheric contribution by taking the coefficients obtained for the day predictants and subtracting the coefficients obtained for the night predictants.

**Figure 9.**
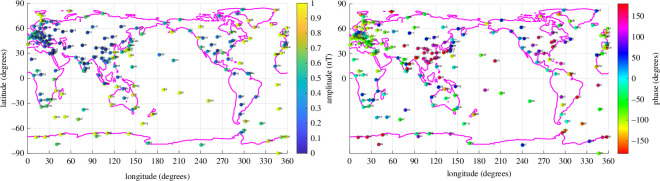
Amplitude and phase of the nighttime ‘oceanic’ $M_{2}$ magnetic tide (for radial component). The higher values in or near the ocean implicate ocean tidal sources. (Amplitudes above 1 nT are drawn to run off the colour scale.)

First, recall that the goal of fitting the nighttime data was to fit the oceanic tidal component. A strong indication that this has been largely accomplished is seen in the geographic distribution of the nighttime fits shown in figure 9. The results plotted here are for the time-harmonic (case 2), but plots using the results from the ephemeris regressions (model 3) look similar. Amplitudes clearly tend to be higher in the ocean basins. In fact, they are also significantly correlated with results from both forward models of the tidal magnetic fields as well as extractions of the $M_{2}$ fields from strictly satellite magnetic data. Here, however, we avoid validations using these references as they have unclear uncertainty levels and insufficient resolution to predict the fields at an observatory location. Instead, we seek support from the land observatory data itself.

**Figure 10.**
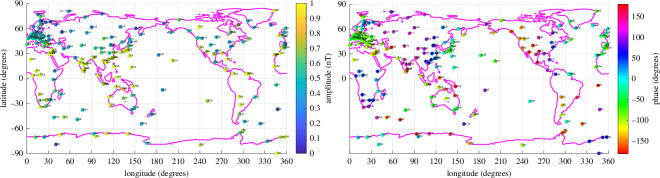
Amplitude and phase of day-night ‘ionospheric’ $M_{2}$ magnetic tide (for radial component)

**Figure 19.**
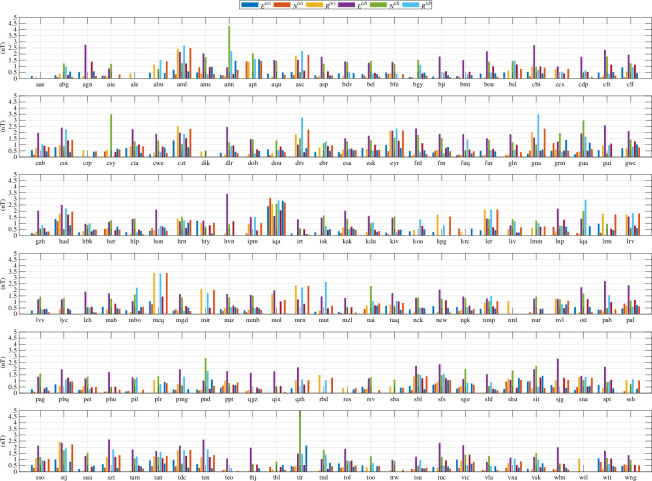
Amplitude of the $M_{2}$ tide extracted from geomagnetic observatory data using lunar ephemeris bases. For each observatory, there are nine estimates representing the three vector component series (*E*, *N*, *R*) using only nighttime data (n), daytime data (d) or night and day data (nd).

**Figure 20.**
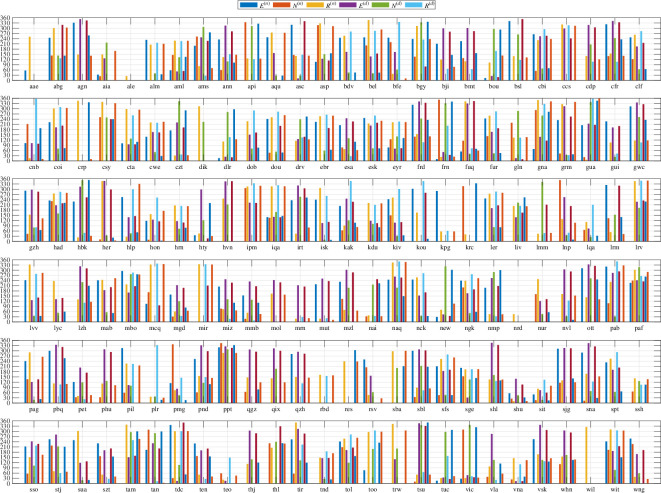
Phase of the $M_{2}$ tide extracted from geomagnetic observatory data using lunar ephemeris bases. For each observatory, there are nine estimates representing the three vector component series (*E*, *N*, *R*) using only nighttime data (n), daytime data (d) or night and day data (nd).

By contrast, the results for the ‘ionospheric’ $M_{2}$ component (taken to be represented by the day minus night coefficients, as described above) are shown in figure 10. Evident here is a correlation with latitude rather than the ocean basins.

Finally, in geomagnetic studies it has often been claimed or assumed that the ionospheric tidal magnetic fields are stronger than the oceanic tidal magnetic fields. From the global results here, it is clear that the ocean, rather than the ionosphere, is in fact the primary contributor to the $M_{2}$ tidal signals in at least the nighttime data. Moreover, similar figures of the geographic distribution of $M_{2}$, but obtained from night + day data, show that the amplitudes remain correlated with the ocean basins. Quantitatively, the mean $M_{2}$ amplitude in the nighttime dataset of observatories is 0.6693 nT using the time-harmonic base, and 0.6736 nT using the ephemeris base. The slightly larger amplitude fit with the ephemeris base is expected because the more physical ephemeris base implicitly fits more of the tidal variance than just the $M_{2}$ time-harmonic base. The amplitudes for the day minus night data are 0.7482 nT for the time-harmonic base, and 0.7009 nT for the ephemeris base.

## 4. Summary of results and conclusions

This study has examined both lunar and solar tidal signals in the collection of land geomagnetic observatories. The focus here in the main paper is on highlighting the most important results for the lunar tidal signals, but further details, results and conclusions are described in appendix A. It is important to note that the methodology here is aimed at a simple and systematic approach that can be applied to each in the global set of observatories. More optimal approaches may be designed for regional studies, and certainly studies involving fusion of data from multiple observatories. We summarize statistical results from the global set. In the long records at many observatories, the p-value of the tidal fits is typically zero or so small that the null hypothesis can clearly be rejected. To evaluate model fits, we compare the ‘adjusted *R*^2^’ values which, like *R*^2^, aim to describe the amount of variance fit but with a penalty for the number of predictors such that models with different numbers of predictors can be compared. However, in examining the formula for the adjusted *R*^2^ statistic, as well as the results, we see that the difference for long series is very small (*R*^2^ and adjusted *R*^2^ are seen to by typically similar) and it is not clear that the compensation for comparing models with different predictor numbers is adequate. For this reason, we emphasize ‘better’ models only where they outperform other models while using the same or fewer number of predictors.

For fitting ocean lunar tidal signals, the best predictant is the (filtered) radial component, although slightly better fits are obtained if principle-component rotation is first performed. In the latter, principle-component analyses are applied to the three-column (time-differenced and filtered) vector series and the third principle component (which remains nearly radial) is used as the predictant. The trade-off for the better fit is less convenience in comparing with other data or model results as the rotation matrices at each observatory must be stored and used for the comparison. The simplest descriptions and comparisons can stay (as in the figures here) with using the radial component.

The best predictors use ephemerides rather than time-harmonic bases. Compared to using the two predictors representing the $M_{2}$ time-harmonic constituent, for example, two predictors formed from the sine/cosine of lunar azimuth (weighted by inverse-cubed lunar distance) fit more tidal variance with higher confidence. This is because $M_{2}$ is only an approximation to the orbit described accurately by the ephemeris coordinates. The ephemeris implicitly fits then $M_{2}$ as well as $N_{2}$ and other semidiurnal constituents. The differences in the fits are, however, relatively small as $M_{2}$ is a good approximation. (Note that the solar ephemeris is also useful for lunar fits as it can be used, as in this study, for calculating the angle from solar zenith and therefore night/day data selection criteria.) The disadvantage of fits using ephemerides is indirect comparison with traditional time-harmonic tidal studies and results from frequency-domain forward models of the tidal magnetic fields.

When comparison/validation with time-harmonic tidal constituents is not required, the ephemerides bases can fit substantially more tidal variance than time-harmonic bases and with the same number or fewer of predictors. While this is not in the main theme of this paper, it is described within the collection of regression models described in detail in appendix A. The most physically based predictors use the ephemerides and gravitational theory to calculate the tidal potential and forces at each observatory location and use these (and their quadrature series obtained through a Hilbert transformation) as predictors. (Note that while only the gradients of the gravitational potential appear in the fluid momentum equation, a closed governing equation that combines the momentum and mass conservation equation shows second-order differentials and therefore the forcing on the flow depends on both the gradients and the central value of the potential.) Indeed, significantly more variance is fitted, as would be expected, but with one interesting caveat. While one would expect that the combined lunar + solar potential (and forces) would provide the ideally best fits, this is not systematically found among the observatories nor even true among their average. A better fit assigns as predictors the lunar and solar potentials/forces separately. If the tidal magnetic fields were only driven/affected by gravitational fields, then the combined lunar + solar approach should indeed be best because of the reduced number of predictors in describing more complete forces. But combining the lunar potential with solar introduces periodicities shared by the non-stationary solar radiation and ionospheric conductivity. Hence, the historical focus on the lunar tides for isolating ocean tidal signals has this simple advantage.

The fits of ocean lunar tidal signals tend to be better near ocean regions with strong tides flowing along gradients of bathymetry and the radial component of the main field. This can be expected from the form of the forcing term in the motional induction equation. But there appear to be disproportionately better fits at island observatories (and outside of auroral latitudes). The highest adjusted *R*^2^ values appear for island observatories, even ones where the tidal magnetic fields are not particularly strong. This may be due to less competing noise arriving from externally induced fields and their concentration along continental coastlines or, more simply, the longer records typical at island observatories (owing to their historical priority in improving global coverage.)

## Figures and Tables

**Figure 11.**
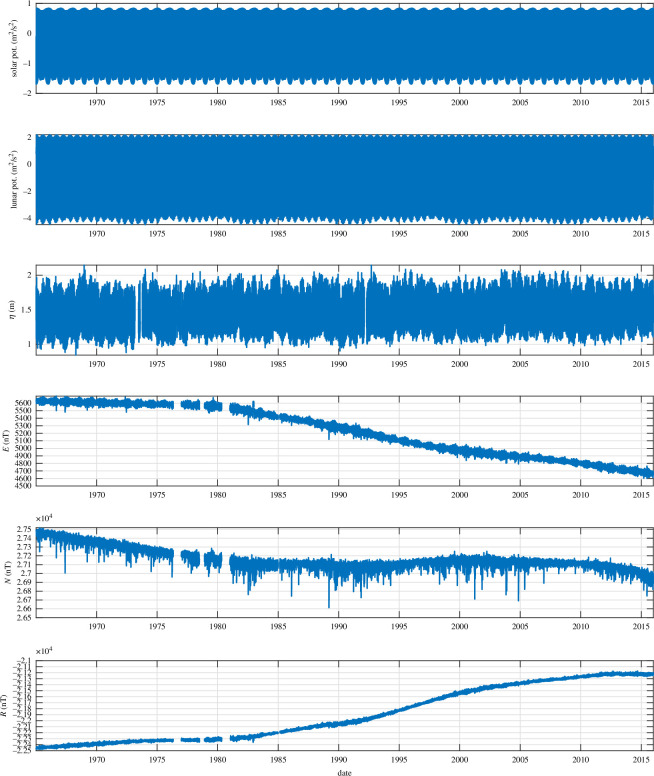
Time series of the solar and lunar gravitational potential, sea level (η), and each of the E,N,R, vector components of the observed geomagnetic field at Honolulu.

**Figure 12.**
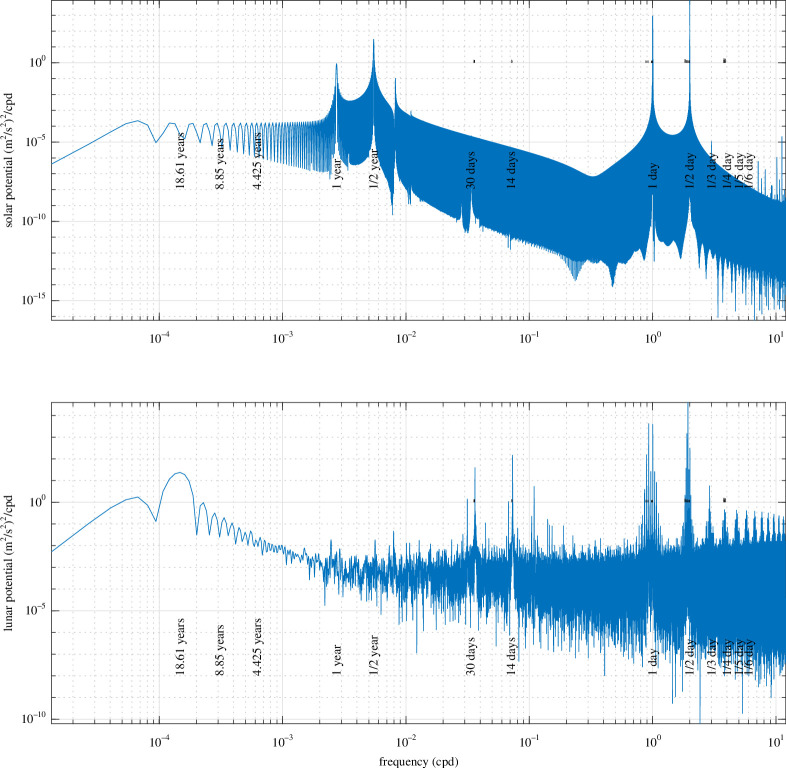
Spectra of solar and lunar tidal gravitational potential at Honolulu.

**Figure 13.**
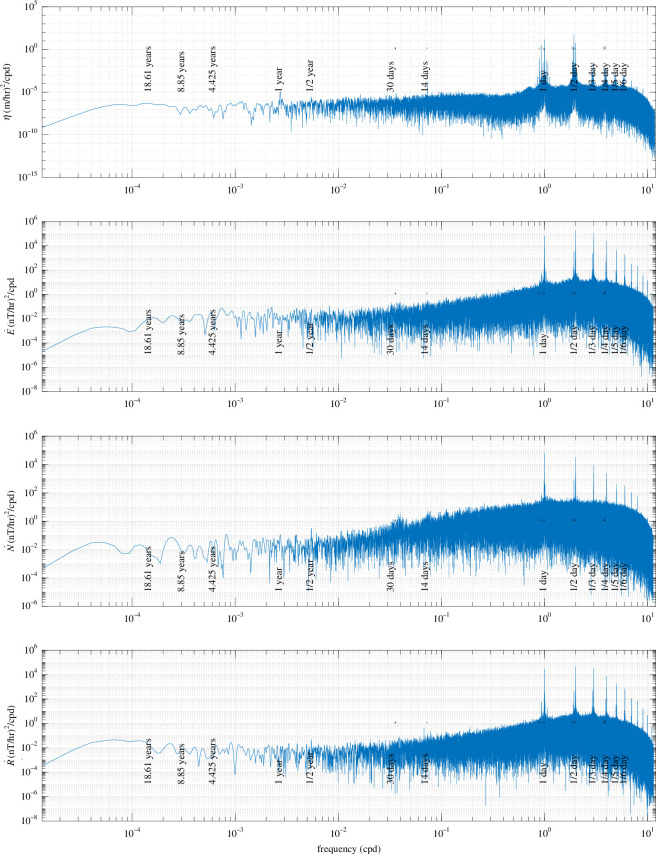
Spectra of time differenced sea level ($\dot{\eta }$), and geomagnetic components ($\dot{E}$, $\dot{N}$, $\dot{R}$) at Honolulu.

**Figure 14.**
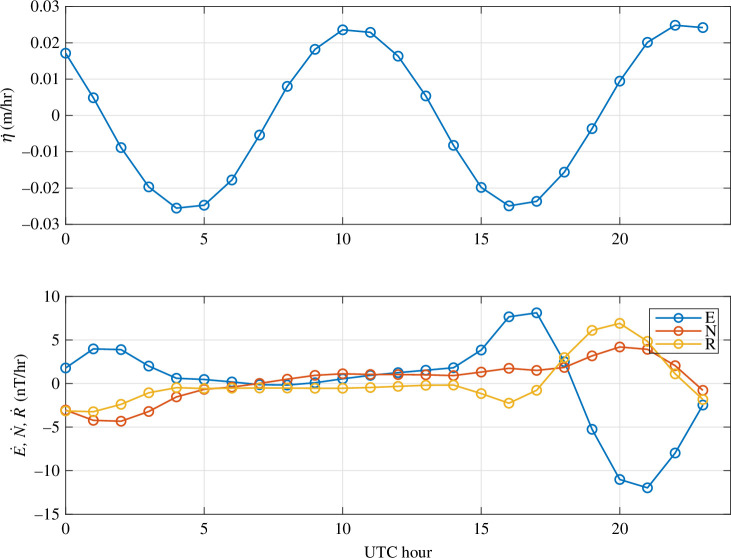
Mean daily variation in sea level ($\dot{\eta }$), and geomagnetic components ($\dot{E}$, $\dot{N}$, $\dot{R}$) at Honolulu. While the sea level shows a clear solar semidiurnal waveform, the waveform for the magnetic data is more complex owing to the effects of radiation.

**Figure 15.**
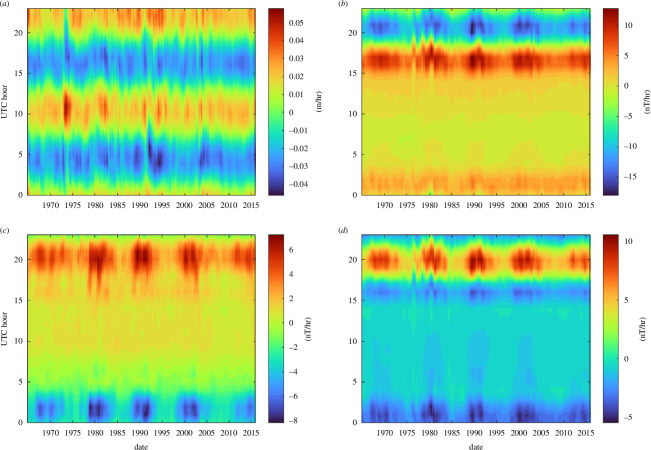
The time modulated solar daily signals for sea level (*a*) and for the geomagnetic components eastward (*b*), northward (*c*) and radial (*d*) removed in step 3 of data processing.

**Figure 16.**
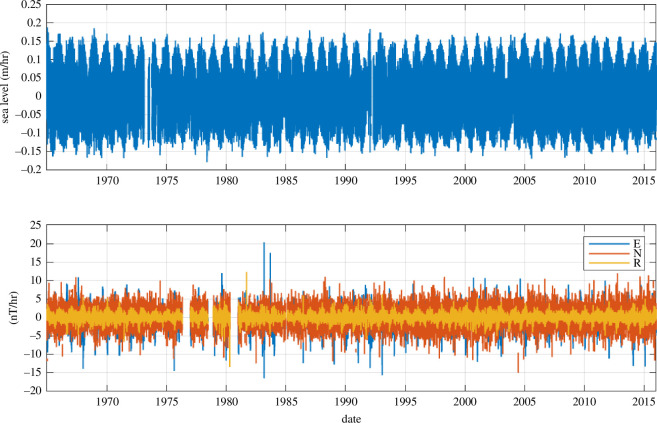
Time series of filtered time-differenced sea level (${\dot{\eta }}_{f}$) and geomagnetic components (${\dot{E}}_{f}$, ${\dot{N}}_{f}$, ${\dot{R}}_{f}$) at Honolulu.

**Figure 17.**
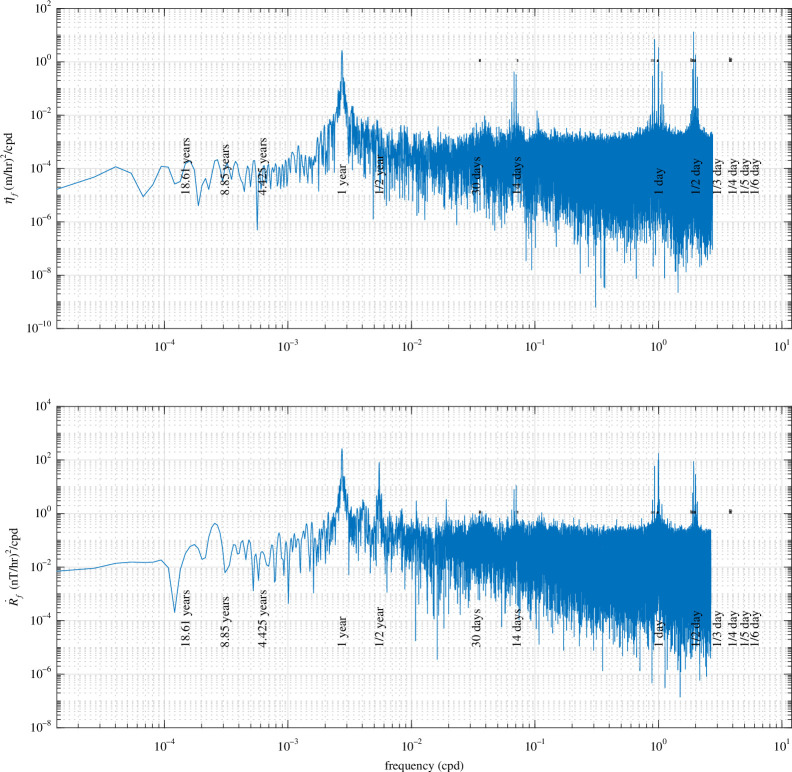
Power spectra filtered time-differenced sea level (${\dot{\eta }}_{f}$) and radial geomagnetic component (${\dot{R}}_{f}$) at Honolulu.

**Figure 18.**
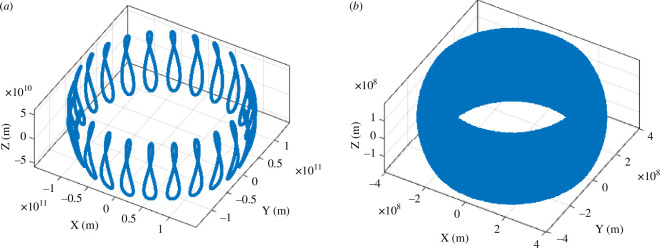
Dots are plotted at the positions (relative to a central Earth-fixed Cartesian frame) of the Sun (*a*) and Moon (*b*) for each time of the hourly sampled geomagnetic data.

**Figure 21.**
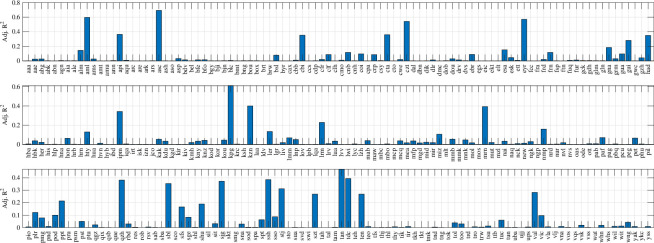
Adjusted *R*^2^ values for the nighttime ‘oceanic’ tidal *M*_2_ coefficients describe the fraction of variance explained in the model.

**Figure 22.**
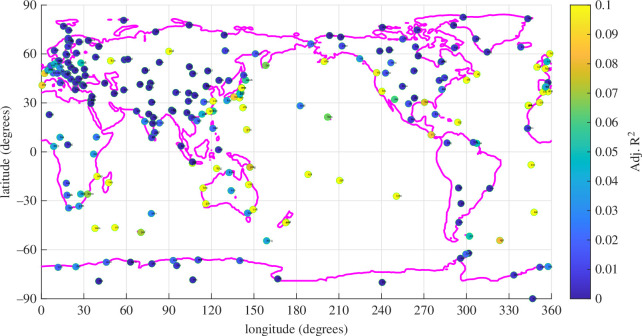
Map of data in figure 21 shows locations of observatories with highest expected $M_{2}$ signal-to-noise ratio in the data. (Values above 0.1 run off colour scale.)

**Figure 23.**
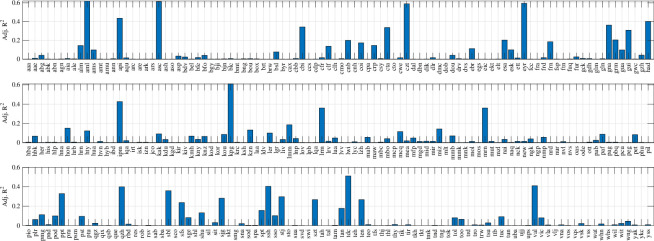
Adjusted *R*^2^ values for the PCA-rotated nighttime ‘oceanic’ tidal $M_{2}$ coefficients describe the fraction of variance explained in the model.

**Figure 24.**
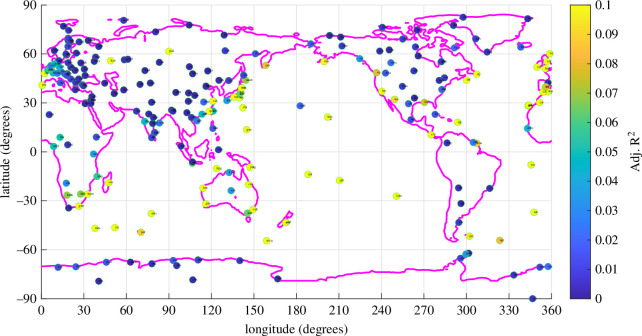
Map of data in figure 23 shows locations of observatories with highest expected $M_{2}$ signal-to-noise ratio in the data. (Values above 0.1 run off colour scale.)

**Figure 25.**
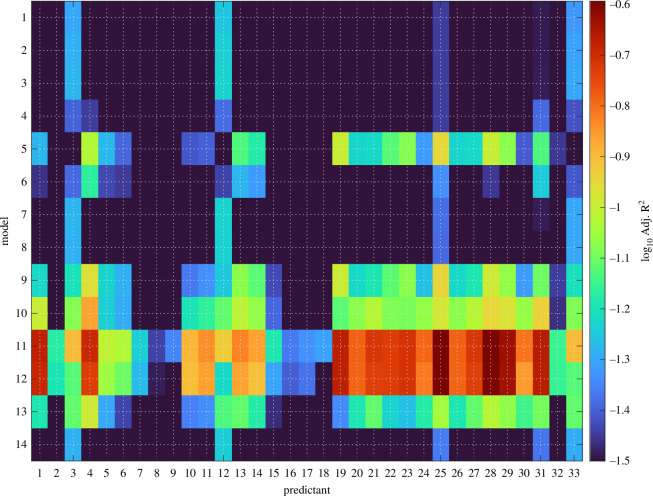
The mean (over all observatories) adjusted *R*^2^ value (Log_10_ scale) for each model/predictant combination. The primary predictant of interest in this study is #3 , representing the nighttime radial magnetic component ${\dot{R}}_{f}$, or its rotated version (#12). The simple two-predictor ephemerides model (model 3) has a slightly higher *R*^2^ than the 10-predictor Chapman–Miller model (model 14). Rotation is also seen to improve the fit of the radial component. model 11 (with the most predictors) has the highest *R*^2^ of the models.

## Data Availability

This study uses publicly available geomagnetic data. The analyses methods of the data are described in the paper. Software from other parties that were used are publicly available at the links given. Geomagnetic data is available at https://wdc.bgs.ac.uk/index.html.

## Footnotes

^1^
The term ‘conductance’ is traditional but has often been used when the depth integral extends not just through the ocean but also the wet, conductive sediments. To avoid ambiguity and also gain symmetry with the terms for other ocean integrals such as ocean heat content (OHC) and ocean salt content (OSC), the term OCC has been introduced in [7].

## Appendix A.

### A.1. Data processing (with example at Honolulu)

To illustrate a specific example of the data processing that are applied to the global set of geomagnetic observatory data, we consider here series from the Honolulu geomagnetic observatory (202.00° longitude, 21.32° latitude). At Honolulu, there is also a tide-gauge station (202.13° longitude, 21.31° latitude) that measures fluctuations in sea level. Both the geomagnetic series (obtained from the World Data Centre for Geomagnetism in Edinburgh, https://wdc.bgs.ac.uk/index.html) and the sea-level series (obtained as ‘research quality’ dataset from University of Hawaii Sea Level Center, uhslc.soest.hawaii.edu) have hourly sampled records extending back over a century. Here, as for the other geomagnetic observatory records over the globe considered in this study, only data within the 50-year time span (1965–2015) are included. Tidal signals in these long Honolulu records have also been well studied (e.g. [30]).

In addition to the geomagnetic and sea-level time series, the time series for the solar and lunar tidal gravitational potential are calculated for the location of Honolulu. Here, ‘tidal gravitational potential’ refers to the components of the gravitational potential of the body (Sun or Moon) that has time-dependence as well as non-uniform gradients over the spherical Earth’s surface. While simpler formulations are available that approximate the potentials using only the lowest-order tidal terms in the McLauren expansion, here the complete tidal potentials are calculated to include the higher-order terms. Specifically, the full potential is calculated and from this are subtracted only those terms responsible for maintaining the orbits but which do not provide forces that are differential in both space and time (the details of this formulation are shown in [31]). This approach is only slightly more accurate than the simpler approaches based on using only the degree-two expansion term. Also used in this study are the time series for the $K_{p}$ geomagnetic disturbance index (obtained from https://doi.org/10.1029/2020SW002641), and the local angle of the Sun from zenith (calculated using the solar ephemeris data). For calculating the potentials and the zenith angle, the required ephemerides series are generated using the SPICE software (obtained from https://naif.jpl.nasa.gov/naif/toolkit.html).

Shown in figure 11 are the time series for the solar and lunar tidal gravitational potential at Honolulu. A plot of the power spectral density for these series is shown in figure 12. (All spectra presented in this study are power-spectral densities calculated using the Lomb–Scargle method, which allows for missing data.) While these results are specific to the Honolulu location, they demonstrate many of the known basic periodicities associated with the Earth’s rotation with respect to the Sun or Moon, the annual period associated with the Earth’s orbit around the Sun, the monthly period of the Moon’s orbit around the Earth, and finally the much longer periods of the lunar perigee describing the variable Earth–Moon distance (8.85 years), and the precession of the lunar nodes (18.61 years) describing the points where the lunar orbit crosses the Earth’s equatorial plane. While not required for the discussion, labels indicating dominant tidal constituents can be seen when magnifying the plot (they are printed near the power level of $10^{0}$). For convenient reference, the ‘dominant’ tidal constituents are selected to match those currently included in the global OSU TPX9.0 ocean tide model (available at https://www.tpxo.net).

Also shown in figure 11 are the time series for the sea surface (η) obtained from the Honolulu tide gauge, and the eastward (E), northward (N) and radial (R) components of the magnetic field observed at the Honolulu geomagnetic observatory. The sea-level shows both high-frequency variability as well as longer periodicities, including an upward trend over the last century.

The geomagnetic time series in figure 11 are dominated by very low frequency ‘secular variation’ associated with processes in Earth’s dynamo core. These secular variations are not of interest in this study. Note that at higher frequencies, the variability in the radial component appears weaker than that of the horizontal components. This would not be expected for ocean tidal magnetic fields and immediately suggests that the variability is dominated by non-oceanic processes. Indeed, the variability is dominated by magnetic fields associated with electric currents in the upper atmosphere (ionosphere) and magnetosphere. In as much as these electric currents flow in large-scale horizontal sheets overhead, the associated magnetic fields observed below are horizontally directed, explaining the stronger variance in E, N. To reduce the influence of this external variability, the component R has been preferred in studies of oceanic magnetic signals. Of course, the Earth is not truly spherical nor concentric with the shell of current flow overhead, and so R is also affected by the overhead currents. A signal strength improvement can be made as in [28], where a principle-component analyses is performed on the vector dataset. The result is to rotate the magnetic vector such that the new axes describe sequentially decreasing fractions of the variance. At Honolulu (and also typical at other observatories), the vector component with the least variance is found to be approximately radial and presumably more perpendicular to the overhead current sheets than is R.

Shown in figure 13 are the spectra for the three (time-differenced) geomagnetic components. For comparison, the spectra for the sea level are also shown. While the sea-level spectra show peaks predicted by the spectra of the tidal gravitational potential (figure 12), the geomagnetic spectra are dominated instead by peaks at harmonics of a solar day. The reason for this is that the sea-level tides are gravitationally driven but the geomagnetic ‘tides’ are also (or even primarily) driven by solar radiation, which strongly affects the atmospheric tides as well as the conductivity of the upper atmosphere.

This distinguishing feature of the geomagnetic data has presented a challenge in extracting the signals owing to gravitational tides even when, as in this study, focussing on the lunar rather than solar tides. One early approach (as in [19]) continued into the modern studies of tidal magnetic signals has been to exclude geomagnetic data taken during the day time, and also to exclude data taken during geomagnetically disturbed times, indicated by indices such as the $K_{p}$ index. Indeed, for the regressions of this study we will not fit the raw series E,N,R but rather a filtered form (${\dot{E}}_{f}$, ${\dot{N}}_{f}$, ${\dot{R}}_{f}$) produced through the following sequence of steps.

1. *Time difference*: apply a central finite-difference operator to each of the series E,N,R and detrend each (i.e. remove mean and slope.)
2. *Remove storm-time data*: replace with NaN (used to represent missing data) observations taken at times when $K_{p}>2$.
3. *Remove the solar daily signal*: because Universal Time Convention (UTC) hourly data is provided, this is easily done by reshaping each of the magnetic series into a matrix of 24 columns (one for each hour) and removing the mean from each column. Because of the long time span of some of the geomagnetic records and the modulation of the daily solar signal with sunspot cycles, a slightly more sophisticated approach is taken here, whereby instead of just removing the column mean, we remove a 1-year-window moving average from each column. One may alternatively reduce the solar daily signal over the long time span by performing a PCA on the time versus hour matrix described above and then reconstructing the data after removing the first and second principle components (which describe the solar daily variation with amplitude and phase changes over seasons/time).
4. *PCA rotation*: (as an option) conduct principle component rotation of the magnetic vector data.
5. *Night/Day data selection*: replace observations taken during day time with NaN. Specifically, the solar ephemeris data and observatory location are used to calculate for each observation time the angle ψ of the Sun from zenith using solid-angle trigonometry. On a spherical Earth’s surface, a point Sun has set below the horizon when $\cos\;\psi <\cos\left(90^{\circ }\right)$. To allow for the non-spherical Earth, and refraction of sunlight, the criterion for ‘nighttime’ is defined as $\cos\;\psi <\cos\left(100^{\circ }\right)$. For comparison, create a similar dataset for ‘day time’ data using the criterion $\cos\;\psi >\cos\left(80^{\circ }\right)$, and also include the data set without removal of day or night data. This replaces the original three-column data with nine columns: $\left[{\dot{E}}_{f},{\dot{N}}_{f},{\dot{R}}_{f}\right]$, $[{\dot{E}}_{f},{\dot{N}}_{f},{\dot{R}}_{f}{]}^{\left(\text{day}\right)}$, $[{\dot{E}}_{f},{\dot{N}}_{f},{\dot{R}}_{f}{]}^{\text{(day+night)}}$, where the first set is the set using only nighttime data, the second uses only day time data,and the last is the data with no removal of day or night data. We also include rotated (following step 4) and unfiltered series for comparison, bringing the total number of geomagnetic predictants in the solution set of this study to 33 (see text below for details).
6. *Weight for secular variation*: when, as in this study, using the geomagnetic series from step 5 as predictants in regressions using data with long time spans, first multiply each of the geomagnetic series by $\tilde{R}$ to compensate for secular variation (see appendix A.2).

These processing steps are used to generate 33 predictant series for the regression analyses at each observatory. Predictants 1–9 are the night, day and night + day, series as described in step 5. Predictants 10–18 are the same as 1–9 but with PCA rotation (the optional step 4). For comparison, these are followed by additional predictants for data with less processing (only step 1 (19–21), steps 1 and 4 (22–24), steps 1 and 2 (25–27) and steps 1, 2, 4 (28–30)). With all of these predictants, the series have been weighted following step 6. Finally, for comparison a version of predictants 1–3 without this weighting is also included (30–33). The predictants most important in this study aimed at separating ocean tidal signals are the series for nighttime vertical (3) and rotated vertical (12) magnetic components.

An example of the solar daily signal removed in step 3 is now described. As shown in figure 14 is the mean UTC daily variations in the Honolulu sea-level and magnetic series. While the sea-level shows a dominant sinusoidal semidiurnal waveform, the magnetic data are more complex and have much larger amplitudes during the day. The sea-level waveform demonstrates the expected gravitational solar semidiurnal tide of the oceans, while the magnetic data demonstrate the expected ‘solar-quiet’ variation owing to solar radiative/ionizing processes in the upper atmosphere. The moving average filter described in step 3 above removes from the data daily signals similar to these but with modulation over the sunspot cycle (figure 15).

The nighttime series ${\dot{E}}_{f}$, ${\dot{N}}_{f}$, ${\dot{R}}_{f}$ (i.e. the predictants 1–3 generated following the steps 1, 2, 3, 5, 6 above) are shown in figure 16. For comparison, a similarly processed series (${\dot{\eta }}_{f}$) for the sea level is also shown. The spectra for ${\dot{\eta }}_{f}$ and ${\dot{R}}_{f}$ are shown in figure 17. Notice the change to the spectral power (relative to that seen in figure 13) owing to the data selection and filtering. The desired effect in the ${\dot{R}}_{f}$ spectra of reducing the geomagnetic power in the solar harmonics is clear; the lunar tidal M2 power has become the tallest peak in the semidiurnal group. There are, however, considerations [32] when applying spectral methods to series containing large amounts of missing data—as is certainly the case here (in 48% of this time span, $K_{p}>2$, leading to the removal of approximately half of the data). With missing data, the Nyquist frequency no longer describes the maximum frequency resolved in the spectrum and low frequencies appear to be affected as well.

A second issue with time-spectral methods for identifying and extracting tidal signals is that the celestial tidal forces are not, strictly speaking, time harmonic. The familiar tidal constituents (e.g. the dominant semidiurnal M2) are not chosen on physical grounds but rather arrive from the decision to conveniently expand the ephemerides parameters and tidal potential in a series of time-harmonic terms, with any physical interpretation of the constituent following (M2, e.g. can be understood to be the tide owing to a Moon approximating an equatorial, circular orbit). Even an hour following the UTC time standard does not correspond precisely with a regular 15° increment of solar azimuthal solar progression with respect to fixed Earth. This is demonstrated in figure 18 where dots are plotted showing the positions of the Sun and Moon for each time of the hourly sampled geomagnetic data. If a UTC hour corresponded regularly with 15° of solar motion around the Earth, then there would not be the loop patterns seen but rather just a collapsed curve showing the seasonal variation. For comparison, the UTC hours of the data sample a full range of lunar azimuths, as expected.

The point of this digression is to indicate that for fitting tidal signals ephemeris coordinates are more natural base functions to use as predictors than are the time-harmonic sinusoids most commonly used in traditional tidal studies.

### A.2. Fitting tidal signals using time-harmonic and ephemerides predictor bases

The best predictor bases to use depend on the application and goals. If the goal is prediction, or fitting as much variance as possible at a specific observatory, then large sets of predictors may be used provided cross-validation checks are made to avoid overfitting and issues of collinearity among predictors can be addressed.

The primary goal of this study is to produce a description of the global distribution of the coefficients for lunar tidal signals in the land geomagnetic observatory records. The goal is then not solely to fit variance but rather to produce reliable estimates for the coefficients of the tides using a data processing method that can be applied systematically to the global set of observatories. Including more predictors can decrease the confidence in individual coefficients derived in the fit as well as complicate the interpretation of the statistics describing the goodness of the fit, especially when some of the predictors are correlated. For these reasons, a preference is toward fits that can explain tidal variance with few predictors that are clearly uncorrelated. The extreme end of this are fits involving only one predictor (or two series in quadrature). In our approach, we present regressions starting from this end and then consider cases with more predictors. In the work reported here, the fits are made using data available in the 50-year window. Including not only data from earlier times, increases the geographic coverage, but also increases concerns related to baseline shifts in recording, and non-stationarity in the tidal response.

To test for the presence of tidal signals in the geomagnetic data series and extract the corresponding coefficients for the constituents, we use the software package UTide [33]. We also extract tidal signals using a second approach which directly uses the ephemerides data describing the location of the Sun and Moon. Both methods are regressions solved using robust iteratively reweighted least squares. They take as input a ‘predictant’ time series of data and fit coefficients for a set of ‘predictor’ base functions. In the UTide case, the predictors are time-harmonic bases for tidal constituent frequencies. In the second approach, cosine and sine functions of the azimuthal ephemerides coordinates are used as base functions. In the second approach, bases explored also include the gravitational potentials calculated from theory using the ephemerides data. The ephemerides is also used to calculate the local solar zenith angle which is used both as a night/day categorical predictor and in generating a radiation function.

For either approach, the predictants are the filtered geomagnetic series described in appendix A.1. The motional induction sources of the ocean tidal magnetic fields depend on the radial component of the main magnetic field (R) as well as its horizontal gradients. While the secular variation in R is typically small enough to ignore (as can be seen in figure 11, it is approximately 6% over the 50-year span at Honolulu), it can be easily compensated for by multiplying the predictants by a non-dimensional weight ${\tilde{R}}^{-1}$. Here, we define $\tilde{R}=R/R(t_{F})$, where $R(t_{F})$ is the value of R at the final time $t_{F}$, which is taken to be the end of 2014 (or the latest previous time in which R was recorded). With this choice for $\tilde{R}$, the tidal coefficients extracted are accurate for present/end times without adjustment. For better estimates for the coefficients for earlier times, t, the coefficients can be multiplied by $(R(t)/R(t_{F}){)}^{-1}$. If the tidal magnetic signals extracted indeed depend on R, as is expected if they are generated by ocean tides, then this weighting of the data by ${\tilde{R}}^{-1}$ should also make the fit more robust (we demonstrate this is indeed the case below).

Secular variation in the gradients of $\tilde{R}$ can also potentially affect the coefficient estimates. The time history of these gradients cannot be determined from the data from the single observatory, and time-dependent geomagnetic field models or other descriptions of the gradients probably do not include high enough spatial resolution to motivate further consideration at present on approaches for weighting for the time dependence of the R gradients.

#### (i) Time-harmonic-base-tidal

UTide requires as input a description of the tidal constituents to fit. Each tidal constituent (e.g. $M_{2}$) involves two predictor series: one representing the cosine of the periodic phase (e.g. cos⁡(ωt), where ω is the frequency of the constituent and t is time), and the other representing the phase-shifted ‘quadrature’ sine series (note that UTide performs nodal corrections for long time series). UTide allows that the constituents be either specified in a list, or they can be automatically selected following a decision tree.

UTide was used in this study to fit each of the predictants for three different input predictor cases:

— Case 1: only the $M_{2}$ bases as predictors;
— Case 2: all of the ‘dominant’ tidal harmonic constituents (specifically, those provided in the OSU Tpx9.0 tidal model which include nine primary ($M_{2}$, $S_{2}$, $N_{2}$, $K_{2}$, $K_{1}$, $O_{1}$, $P_{1}$, $Q_{1}$, $S_{1}$), two long period ($M_{f}$, $M_{m}$) and four nonlinear ($M_{4}$, $M_{S4}$, $M_{N4}$, $M_{N2}$) harmonic constituents).
— Case 3: a decision tree automatically selects the constituents that can be retrieved.

#### (ii) Ephemerides base tidal fits

For each of the 33 predictants, we consider here a range of 14 iteratively reweighted robust linear regression models involving different predictor functions based on the lunar and solar ephemerides, the tidal gravitational potential and sea-level data. We are interested in (i) predictors that give results that can be compared to the M2 estimates in the harmonic fits above, (ii) predictors capable of explaining the most variance with the fewest predictors and (iii) larger sets of predictors that explain more variance and yet remain physically based. We treat these topics sequentially below.

In the time-harmonic bases, each constituent involves two predictor series ($M_{2}$, e.g. has the predictors [$\cos(\omega_{M2}t$), $\sin(\omega_{M2}t$)], where $\omega_{M2}$ is the $M_{2}$ frequency (with a period of approximately 12.42 h) and t is time.) The $M_{2}$ bases are essentially a harmonic-approximation to the bases $\left[\cos(2\varphi_{M}),\sin(2\varphi_{M})\right]$, where φ is the azimuthal coordinate of the Moon relative to a frame fixed on Earth. Because $\left[\cos(2\varphi_{M}),\sin(2\varphi_{M})\right]$ track the lunar position (and therefore its gravitational field) more accurately, we expect that for fitting data with lunar tidal signals the predictors $\left[\cos(2\varphi_{M}),\sin(2\varphi_{M})\right]$ should fit slightly more variance than do the predictors $\left[\cos(\omega_{M2}t),\sin(\omega_{M2}t)\right]$. Because the tidal forces vary with the inverse radius cubed, we expect an even better set of two predictors is $\left[\cos(2\varphi_{M}){\tilde{r}}_{EM}^{-3},\sin(2\varphi_{M}){\tilde{r}}_{EM}^{-3}\right]$, where ${\tilde{r}}_{EM}$ is the non-dimensional time-dependent distance between the Earth and Moon centres. (We choose ${\tilde{r}}_{EM}$ to be the distance divided by its time mean.) As will be seen in the results, these three models indeed show a sequence of improved tidal fits, as expected. We will also see that without the ${\tilde{R}}^{-1}$ weighting to compensate for secular variation, the adjusted *R*^2^ values in each of these three models for ${\dot{R}}_{f}$ drops slightly.

Results from predictor model 3 are also compared with the M2 coefficients extracted using UTide. UTide makes nodal and satellite corrections in its extraction of M2. While it is not obvious that these should recover the same corrections implicit in the use of the ephemerides predictors $\left[\cos(2\varphi_{M}){\tilde{r}}_{EM}^{-3},\sin(2\varphi_{M}){\tilde{r}}_{EM}^{-3}\right]$, it is demonstrated (figure 8) with the global set of magnetic data that the tidal fit for UTide $M_{2}$ and the tidal fit from model 3 provide nearly identical estimates. This match between tidal signals extracted using the two different methods provides useful cross validation.

While model 3 provides the best two-predictor model that can be compared with fits to the time-harmonic $M_{2}$ bases, there might there be other sets of two predictors that explain even more variance in the predictants. Confidence levels in results do not account for the so-called ‘file-drawer’ bias whereby negative or uninteresting results are not reported, and there is indeed a wide range of predictor series that could be tried and discarded if the results were found to be uninteresting. To avoid the file-drawer bias, here we consider only predictors that are physically based and we perform regressions systematically for all predictants.

In considering the physical motivation for predictors, first consider that the gravitational tides are more fundamentally related to the time-dependent tidal gravitational potential than they are to the ephemeris parameters. Specifically, the tides depend on both the horizontal gradient and second-order spatial derivatives of the potential field. (While only the gradient appears in the momentum equation, the momentum equation is not closed. Combining the momentum equation with the mass-conservation/continuity equation to create a single governing equation for the pressure shows terms involving second-order derivatives in the potential.)

For regression model 4, we define lunar tidal potential $\Phi_{Mtide}$ to be the first predictor. The tides respond to $\Phi_{Mtide}$ but need not be in phase with it. Therefore we assign as the second predictor $\Phi_{Mtide}^{Q}$, which is a quadrature shifted version of $\Phi_{Mtide}$, which we obtain as the imaginary component of the Hilbert transform of $\Phi_{Mtide}$. Model 5 is similar but for the solar gravitational potential, and model 6 combines both lunar and solar potentials. For fitting strictly gravitational tides, one should expect that the combined potential will fit more variance as there is no difference in the nature of the force whether it be from the Sun or Moon. However, including the solar potential also introduces the periodicities of the radiational effects and so a better fit is not systematically achieved over using just the lunar potential as bases.

More variance in the predictants can potentially be explained using more than two predictors but comparing model fits with the fits from models using only two predictors must be done carefully. While model fits using the same number of predictors can be rated by the *R*^2^ statistic describing the fraction of variance explained, the adjusted *R*^2^ statistic attempts to make the models more inter comparable by creating a penalty for additional predictors.

In designing models with more than two predictors, consider that the Laplace tidal equations governing the tidal flow show that the tides are forced by both the gravitational potential and its gradients. Including the gradients increases the number of predictors from two to six. Further, while there is no spherical-harmonic degree 1 form in the tidal gravitational potentials, the cosine of the solar zenith angle (cos⁡ψ) and its quadrature series could fit the lagged effects of radiation and pressure on the Earth’s magnetosphere by the solar wind. What we refer to here as the radiation function $F_{rad}$ is the time series of cos⁡ψ (inversely weighted by the solar distance) with values cos⁡ψ<0 replaced by zero. A quadrature series $F_{rad}^{Q}$ is similarly calculated from the Hilbert transform of cos⁡ψ.

Model 7 (with four predictors) is the same as model 3 but it allows for modulation by $F_{rad}$ (i.e. the predictors in model 3 are multiplied by 1 + $F_{rad}$). Model 8 (with six predictors) is model 7 with the modulation extended to $1+F_{rad}+F_{rad}^{Q}$. Model 9 (four predictors) includes as predictors the series for the lunar and solar potentials (and their quadrature series) represented individually. Model 10 (eight predictors) includes predictors for the combined lunar + solar potentials and their horizontal gradient (forces), and predictors for a degree−1 term (cos⁡ψ) and its quadrature are also included. Model 11 (14 predictors) is similar to model 10 but with the lunar and solar potentials and their forces represented individually. Model 12 (eight predictors) is similar to model 10 but using only the solar potential and forces rather than the combined. Model 13 (eight predictors) is similar to model 12 but for the lunar potential and forces. Finally, model 14 (10 predictors) uses the predictor bases of the Chapman–Miller method described in the next section. The predictants and models are listed in tables 1 and 2.

**Table 1.** List of predictants used in the study, grouped by their vector components.

predictant #	description
1–3	nighttime vector components
4–6	daytime vector components
7–9	combined night and day vector components
10–12	PCA-rotated nighttime vector components
13–15	PCA-rotated daytime vector components
16–18	PCA-rotated combined night and day vector components
19–21	unfiltered vector components
22–24	PCA-rotated unfiltered vector components
25–27	filtered and PCA-rotated vector components
28–30	secular-variation weighted vector components
31–33	secular-variation weighted PCA-rotated vector components

**Table 2.** List of models used in the study, involving different sets of predictors for the regressions.

model	description	num. of predictors
1	cosine and sine of M2 phase (i.e. time harmonic)	2
2	cosine and sine of lunar azimuth	2
3	model 2 weighted by lunar distance	2
4	lunar potential (and its quadrature)	2
5	solar potential (and its quadrature)	2
6	lunar+solar potential (and its quadrature)	2
7	model 4 with modulation by radiation function	4
8	model 7 with additional modulation by quadrature radiation function	6
9	individual lunar and solar potentials (and quadrature series)	4
10	combined lunar+solar potentials (and gradient), plus degree−1 term (cos ψ and its quadrature)	8
11	similar to model 10 but with lunar and solar potentials (and gradient) represented individually	14
12	similar to model 10 but using only the solar potential (and gradient)	8
13	similar to model 12 but for the lunar potential (and gradient)	8
14	uses the predictor bases of the Chapman–Miller method	10

#### (iii) Comparison with Chapman–Miller method

In the historical Chapman–Miller method of regression [19], data selection in the geomagnetic predictant series excludes data during geomagnetically disturbed times (e.g. $K_{p}>2$) but otherwise retains data from both night and day. The Chapman phase law,

(A 1)
$$\begin{array}{ccc} & L=\Sigma_{n=0}^{4}l_{n}\sin\left[\left(n-2\right)t_{s}+2\tau +\lambda_{n}\right], & \end{array}$$

models the predictant L using time-harmonic predictor bases involving the solar $t_{s}$ and lunar τ daily mean motion (represented as angular arguments approximating the mean azimuthal coordinates of the bodies with respect to a fixed Earth frame). The 10 coefficients for the amplitude $l_{n}$ and phase $\lambda_{n}$ are fitted in the regression. The oceanic contribution is recognized as the n=2 term as it does not depend on $t_{s}.$ The ionospheric contribution is L with the n=2 term subtracted.

Rewriting equation (A 1) in terms of cosine and sine terms shows that the regression involves 10 predictor series. However, reading through the description in [19] of what physical effects are attempted in the model in equation (A 1) suggests that a more accurate model that also uses fewer predictors can be performed using ephemerides rather than time-harmonic predictor bases.

First, using the lunar and solar azimuth coordinates ($\varphi_{M}$ and $\varphi_{S}$) from the ephemerides data is slightly more accurate than using the steady time-mean motions described in $t_{s}$ and τ. More importantly, the number of predictors in equation (A 1) is a consequence of the inefficiency of using time-harmonic bases to fit the cycle of solar radiation. As described in [19], equation (A 1) attempts to represent the model

(A 2)
$$L=\left(a\;\sin2\tau +b\;\cos2\tau \right)+\left(c\;\sin2\tau +d\;\cos2\tau \right)F_{rad}(t_{s}),$$

where the first parenthesized term represents the oceanic part active during both night and day, and the second parenthesized term represents additional contributions during the day owing to the radiation function $F_{rad}$. If $F_{rad}$ is prescribed, then equation (A 2) involves four predictors. The extra six predictors used in equation (A 1) is then a consequence of the inefficiency of representing $F_{rad}$ with time-harmonic bases involving $t_{s}$. The first part of this inefficiency is that $t_{s}$ can be used to describe day versus night only in an annual-mean approximation. In that case, independent of the latitude or season, $F_{rad}$ is assumed to vary as a 24 h sinusoid (e.g. $\cos\;t_{s}$) with the negative values set to zero. The second part of the inefficiency is that representing such a chopped-off sinusoid function with time-harmonic bases requires multiple harmonic components and therefore leads to more predictors. One expects that using more predictors than necessary to capture the same amount of variance will lead to less confidence in the individual coefficients.

Using ephemerides, by contrast, no harmonic expansion of $F_{rad}$ is required. We can describe (non-dimensional) $F_{rad}$ more directly and accurately as

(A 3)
$$F_{rad}=\cos(\psi_{S}){\tilde{r}}_{ES}^{-1},$$

where $\psi_{S}$ is the solar zenith angle at the time and location of the observations and ${\tilde{r}}_{ES}$ is the non-dimensional distance between the Earth and Sun. This description is accurate for any time of year or location. It is also represented directly with one series and does not require an expansion in multiple series as above. The factor ${\tilde{r}}_{EM}^{-1}$ makes the representation slightly more realistic because of the inverse-distance of the radiation intensity.

Using the ephemerides instead of harmonic bases, it is expected that equation (A 2) can be formulated more accurately as

(A 4)
$$L=\left(a\cos(2\varphi_{M}){\tilde{r}}_{EM}^{-3}+b\sin(2\varphi_{M}){\tilde{r}}_{EM}^{-3}\right)+\left(c\cos(2\varphi_{M}){\tilde{r}}_{EM}^{-3}+d\sin(2\varphi_{M}){\tilde{r}}_{EM}^{-3}\right)F_{rad},$$

where $F_{rad}$ is given by equation (A 3). We see then that only four (rather than 10) coefficients are fitted to four predictor series prescribed with high accuracy by ephemerides data.

Note that while equation (A 4) is expected to be a better regression model than equation (A 2) it can only be expected to be also better than equation (A 1) if the underlying physical arguments on radiation used in formulating equation (A 1) are valid. Indeed, with 10 predictors as opposed to four, equation (A 1) could explain more of the data variance if any processes outside these physical assumptions happen to be fitted. We can here examine this point as the calculated solution set includes cases for both the equation (A 1) and equation (A 4) models. The predictant in this case are the data following only steps 1 and 2 above as both night and day data are required and the solar daily signal should not be filtered out.

The results for Honolulu show that the adjusted *R*^2^ value for the 10-predictor Chapman phase model (equation (A 1)) is 0.110, while the value is in fact slightly higher (0.112) for the four-predictor model in equation (A 4). Given that essentially the same amount of variance has been explained with the models, the equation (A 4) model should be preferable because it has fewer predictors (and therefore fewer fitted coefficients), and because the formulation of the predictors is more direct and physically based.

The slightly higher adjusted *R*^2^ value for equation (A 4) model could be due to the more realistic description of $F_{rad}$. Note that for the comparison, the angular arguments $t_{s}$ and τ in equation (A 1) were in fact replaced with the more accurate ephemerides coordinates $\varphi_{S}$ and $\varphi_{M}$ to focus on only the differences owing to the different representations of $F_{rad}$.

A result we see in the global set of data that regressions with ephemeris predictor bases can fit the lunar tidal results as well or better despite having fewer predictors.

### A.3. Results from global geomagnetic observatories

Hourly data within the period (1965–2015) from 288 land geomagnetic observatories was processed just as described for the case of the Honolulu geomagnetic observatory data (see appendix A.1). That is, for each observatory, 33 geomagnetic predictant series were each fitted to 17 different predictor models (three using the time-harmonic bases in UTide, and 14 using ephemeris and gravitational-potential bases). For clarity, we refer to the three time-harmonic predictor models as ‘cases’ 1–3 to distinguish from ‘models’ 1–14 used to refer to the models with the ephemeris bases.

#### (i) Observed-*M*_2_-1

We consider here the $M_{2}$ tidal constituent. While the $M_{2}$ time-harmonic bases do not capture as much tidal energy as the ephemerides/potential-derived bases, much of the discussion of ocean tidal magnetic fields in observations and modeling has been focussed on *M*_2_. This time-harmonic base is not required to extract tidal signals from the data but it is required in the time-harmonic numerical forward models that predict the magnetic field. Hence, a description of the observed $M_{2}$ constituent is important for multiple reasons.

The $M_{2}$ estimates using time-harmonic bases are directly available from the UTide results (here, we describe case 2, where all the primary constituents are fitted). As described in appendix A.1, it is expected that the ephemerides model 3 is comparable to the *M*_2_ bases and so the results using these ephemerides predictors are used as a cross-check of the UTide $M_{2}$ results. To compensate for the time-differencing and obtain the coefficients reflecting the original (without time differencing) series, we translate the amplitude/phase coefficients to complex form, divide by −iω, where $i=\sqrt{-1}$ and ω is the constituent (*M*_2_) frequency, then convert again to amplitude and phase.

The estimates from the time-harmonic fit are shown in figures 6 and 7. The estimates from the ephemeris fit are shown in figures 19 and 20.

The estimates from the two different bases agree well (see figure 8 for example). Overall confidence in the estimates tends to be high because most of the observatories provide much data within the 50-year window and because only fits with high confidence are admitted. Note that, the time-harmonic fits admit more of the observatories than do the ephemeris fits. This is at least partially due to the differences in the way the admission criteria are posed in each. In the time-harmonic case, the confidence levels (with criterion > 95%) are calculated in UTide using a coloured Monte Carlo method, whereas with the ephemeris fits the criterion is that the p-value must be < 0.5.

#### (ii) Where is the $M_{2}$ signal-to-noise ratio highest?

Previous sections have described the observatory locations where the ocean $M_{2}$ magnetic signals are the strongest. But this does not adequately describe the locations with the highest $M_{2}$ signal-to-noise ratio because the level of confounding noise varies between locations. This is addressed here with the adjusted *R*^2^ values of the model fits. The *R*^2^ value can be understood as representing the fraction of the data variance explained by the model fit. Adjusted *R*^2^ values are used to describe regressions with multiple predictors by imposing a penalty on the number of predictors to avoid overfitting.

Results for the adjusted *R*^2^ values associated with the nighttime *M*_2_ fits (represented here by predictant 3 fit to model 3) are shown in figures 21 and 22. While the results show higher *R*^2^ values tend to follow the locations where the tidal magnetic signals are stronger, there also seems to be a nearly systematic elevation of *R*^2^ values for island observatories. This could be because the well-known ‘coastal effect’ in magnetic data describing the concentration of externally induced electric currents at coastlines (which enter here as competing noise) is not as strong at island observatories. The lowering of *R*^2^ values at higher latitudes is also expected because of increased geomagnetic noise in and approaching the auroral zones.

If instead of the vertical component (predictant 3), we consider the rotated vertical component (predictant 12), the statistical fit improves, as can be seen in figures 23 and 24. Interestingly, the elevation of adjusted *R*^2^ values near the oceans is more complete with the rotated data, indicating that rotation has systematically or at least typically generated a new vertical component with a higher ocean-tidal signal-to-noise ratio. Some exceptions remain: the low values at the island of Midway, northwest of Hawaii, can be explained by the short data record. The low values at Hermanus (South Africa) and a few other observatories may be due to weak tidal magnetic fields at their locations.

### A.4. Summary of models beyond *M*_2_

Over the set of observatories, the mean adjusted *R*^2^ values of all the ephemerides model fits from this study are shown in figure 25. Beyond models aimed at representing *M*_2_, more ocean tidal variance can be fitted with models using more predictors. Moreover, the fits can show higher adjusted *R*^2^ values, indicating higher statistical significance. The best two-predictor model fitting nighttime lunar tides is model 3, which uses the sine and cosine of the lunar azimuth as predictors (weighted by lunar distance). A significantly better fit (according to the adjusted *R*^2^ value) is achieved by model 13, which uses instead the lunar gravitational potential and its gradients (six predictors). An even better fit is achieved by model 11 (12 predictors) which adds similar bases for the solar potential. While the adjusted *R*^2^ attempts to compensate for overfitting with more predictors, conclusive comparisons should be restricted to models with similar numbers of predictors (or where the better fit is found in the model with fewer predictors.)
